# HIF1-α induces mitochondrial fission in trophoblastic cells during early pregnancy and in preeclampsia

**DOI:** 10.1016/j.isci.2026.115674

**Published:** 2026-04-09

**Authors:** Léa Poinsignon, Audrey Chissey, Juliette Colombel, Bertrand Lefrère, Charlotte Izabelle, Lucie Bernard, Jean-Louis Beaudeux, Thierry Fournier, Ioana Ferecatu, Isabelle Hernandez, Amal Zerrad-Saadi

**Affiliations:** 1 Université Paris Cité, INSERM, UMR-S 1139 (FPRM), Faculté de Pharmacie de Paris, 75006 Paris, France; 2 Service Biochimie, AP-HP, Hôpital Necker Enfants Malades, 75006 Paris, France; 3 FHU Prem’IMPACT, Paris, France; 4 Université Paris Cité, Inserm, CNRS, P-MIM, PICMO, 75006 Paris, France

**Keywords:** cell biology, developmental biology

## Abstract

The placenta undergoes a major oxygen transition between 7 and 9 gestation weeks (GW) and 12–14 GW. Inadequate adaptation to these environmental changes leads to oxidative stress and the release of syncytial knots, a hallmark of preeclampsia (PE). As mitochondria play a central role in redox regulation, we investigated mitochondrial network dynamics and the role of hypoxia-inducible factor 1-alpha (HIF1-α) in trophoblasts during early pregnancy and in PE. HIF1-α is stabilized in placenta before 9 GW, associated with mitochondrial hypertubulation and increased mitofusin (MFN) 1 expression. Experimental stabilization of HIF1-α using cobalt chloride induces mitochondrial fission through downregulation of MFN1 and MFN2, and upregulation of dynamin-related protein 1 (DRP1). In pre-eclamptic placentas, significantly elevated mitochondrial fission 1 protein (FIS1) levels suggest enhanced mitochondrial fission, supporting FIS1 as a potential biomarker of PE-associated placental alterations.

## Highlights

• Oxygen transition is associated with HIF1-α degradation and mitochondrial fusion
• HIF1-α promotes mitochondrial fission through downregulation of MFN1
• Late- preeclampsia (late-PE) is associated with increased mitochondrial fission
• FIS1 emerges as a potential biomarker for late-PE

## Graphical abstract

**Figure:**
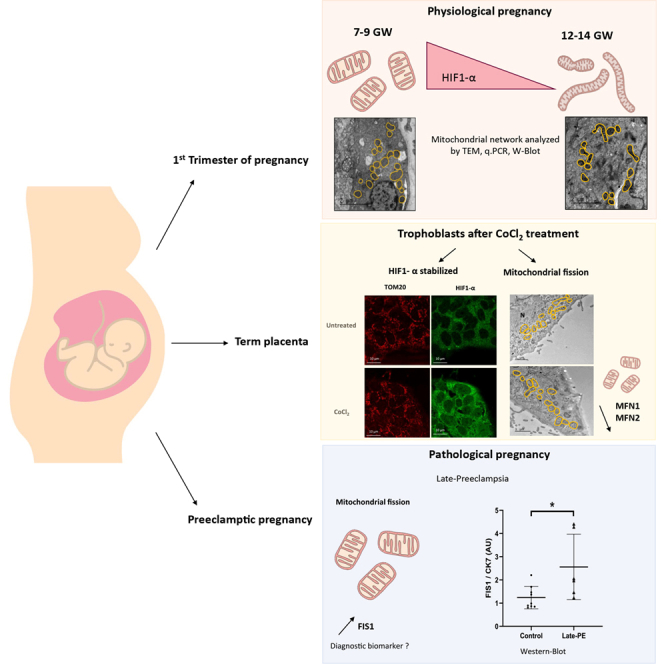

## Introduction

The placenta is a transitional organ, which ensures the fetal development by delivering gas and nutrients to the fetus. Its development, called placentation, begins on day 8 post-fertilization. The blastocyst, resulting from parental gametes fusion, anchors in the uterine mucosa and differentiates at its periphery into trophectoderm. This trophectoderm differentiates into villous cytotrophoblast (VCT) and syncytiotrophoblast (ST), which are the functional cells of the human placenta. VCTs and ST are localized at the periphery of the chorionic villi, bathing in the intervillous chamber in contact with the mother’s blood. Chorionic villus represents the structural and functional unit of the human placenta, responsible for feto-maternal exchanges but also the endocrine function by secreting beta-human chorionic gonadotropin (β-hCG) and pregnancy-associated plasma protein-A (PAPP-A), and the angiogenic function by secreting placental growth factor (PlGF).^1^^,^^2^

During early pregnancy, between 9 and 12 gestation weeks (GW), the placenta experiences significant environmental changes. The partial O_2_ pressure (PaO_2_) in intervillous chamber increases progressively from 20 to 55 mmHg, corresponding to the removal of trophoblastic plugs from maternal spiral uterine arteries.^3^^,^^4^ Low PaO_2_ during the first GW limits the production of reactive oxygen species (ROS), which can be harmful during embryonic organogenesis, and promotes the stabilization of the transcriptional factor hypoxia inducible factor 1-alpha (HIF1-α).^5^ HIF1-α further enhances the placental angiogenesis (PlGF) and favors the invasion of trophoblastic cells into the maternal myometrium. Oxygen transition during early pregnancy leads to drastic modifications of the redox state into trophoblastic cells, including an increase expression of antioxidant enzymes (superoxide dismutase 1 [SOD1], catalase) after 12 GW,^6^ and a modulation of the main sources of ROS, NADPH (nicotinamide adenine dinucleotide phosphate) oxidase enzymatic complex and mitochondria.^6^^,^^7^^,^^8^

Mitochondria are the metabolic hub of the cell, serving as the site of Krebs cycle and ATP synthesis by the oxidative phosphorylation. These metabolic activities are localized at the mitochondrial inner membrane (MIM) and are modulated according to environmental conditions. Mitochondrial metabolic activity seems to increase during oxygen transition in early pregnancy^9^ but until now few studies have evaluated these mitochondrial mechanisms. Nevertheless, we know that VCTs and ST have distinct mitochondrial phenotypes, which are involved in specific trophoblastic functions. Indeed, mitochondria in VCTs are relatively large (around 0.5 μm) and have an elongated shape with lamellar cristae, whereas mitochondria from ST are smaller (≤0.1 μm), spherical with tubular cristae and a less dense matrix.^10^ The smallest mitochondria from ST are associated with a lower oxidative phosphorylation activity (low ATP synthase) but an increased efficiency of cholesterol transport across membranes, increasing the steroidogenesis and therefore contributing to the endocrine function of ST. In contrast, mitochondria from VCTs assure a high metabolic activity, which permits the renewal of VCTs pool and the fusion process into ST.^10^^,^^11^ These different mitochondrial phenotypes are made possible by the complex structure of the organelle. It is composed of a double membrane—the inner and the outer membrane—which delimit the intermembrane space. These membranes are in constant dynamics alternating between fusion and fission processes, driven by GTPase-dynamin family proteins. On one hand, three main proteins mediate fusion process: mitofusin (MFN) 1 and 2 (MFN1 and MFN2) and optic atrophy-1 (OPA-1). MFN1 and MFN2 are localized at the outer membrane and their GTPase activity induces a conformational change and fusion of the outer membranes via SNARES proteins.^12^^,^^13^^,^^14^ After fusion of the outer membrane, the inner membranes fuse through the GTPase-active protein OPA-1, linked to kinesin. On the other hand, two main proteins mediate fission process: dynamin-related protein 1 (DRP1) and mitochondrial fission 1 protein (FIS1). DRP1 translocate from the cytosol to the mitochondria where it interacts with FIS1 on the inner and outer mitochondrial membranes (OMMs). DRP1 proteins accumulate in a ring around the mitochondrial outer membrane, constrict and fission mitochondrial membranes by interacting with the actin/myosin network.^12^^,^^13^^,^^14^ Mitochondrial fusion and fission depend on energy metabolism and cellular needs (differentiation, proliferation, apoptosis, etc.), mitochondrial membrane potential, and mitochondrial redox status. Fusion is often induced during moderate mitochondrial/cellular stress (ROS production and protein oxidation), enabling the fusion of damaged mitochondria with a healthy one, allows mixing of mitochondrial contents and rescue of damaged mitochondria.^15^ Fission is mainly induced during severe mitochondrial/cellular stress (excessive mitochondrial ROS production, mitochondrial DNA damage, etc.), enabling the elimination of strongly damaged mitochondria either by the degradation system (proteasome) or by mitophagy.^15^

Mitophagy is largely described during preeclampsia (PE), a pregnancy disease occurring after 20 GW. Preeclampsia is characterized by a maternal hypertensive disorder (≥140/90 mmHg) with a proteinuria (≥0.30 g/24 h), or a proteinuria/creatininuria (P/C) ratio above or equal to 30 mg/mmol. Preeclamptic syndrome can be distinguished into two subgroups: early-PE, which begins before 34 GW and late-PE occurring after 34 GW. These two subtypes of PE are distinct in their pathophysiological processes: early-PE often involves a placentation defect whereas late-PE occurs after syncytial damages linked to hypoxia-reoxygenation phenomena leading to ROS production.^16^ These physiopathological processes are associated with mitochondrial damages, generating ROS but also including mitobiogenesis alterations, decrease in metabolic activity, and increase of apoptosis activation.^17^ Mitochondrial dynamic during PE is not yet clearly understood, few studies have evaluated proteins expression of MFN, OPA-1, DRP1, or FIS1 in preeclamptic placentas but results are inconclusive, alternatively favoring fission or fusion processes.^9^^,^^18^^,^^19^^,^^20^

Objectives of this study are thus triple: (1) studying the mitochondrial phenotype in early pregnancy and the changes in mitochondrial dynamics that occur during oxygen transition (between 9 and 12 GW), (2) analyzing the mitochondrial dynamic during early- and late-PE in search of biomarkers of syncytial damages, and (3) understanding the implication of HIF1-α in the modulation of mitochondrial dynamics in trophoblastic cells. Consequently, chorionic villi from 7 to 9 GW, 12–14 GW, and from preeclamptic placentas were observed by transmission electron microscope (TEM), and protein levels implicated in mitochondrial dynamics were analyzed by western blot. Involvement of HIF1-α was then analyzed on primary trophoblastic cell culture exposed to cobalt chloride (CoCl_2_) and observed by TEM and confocal microscopy. Additionally, mitochondrial protein levels were analyzed by western blot.

## Results

### HIF1-α expression decreases at 12–14 GW

**Figure 1:**
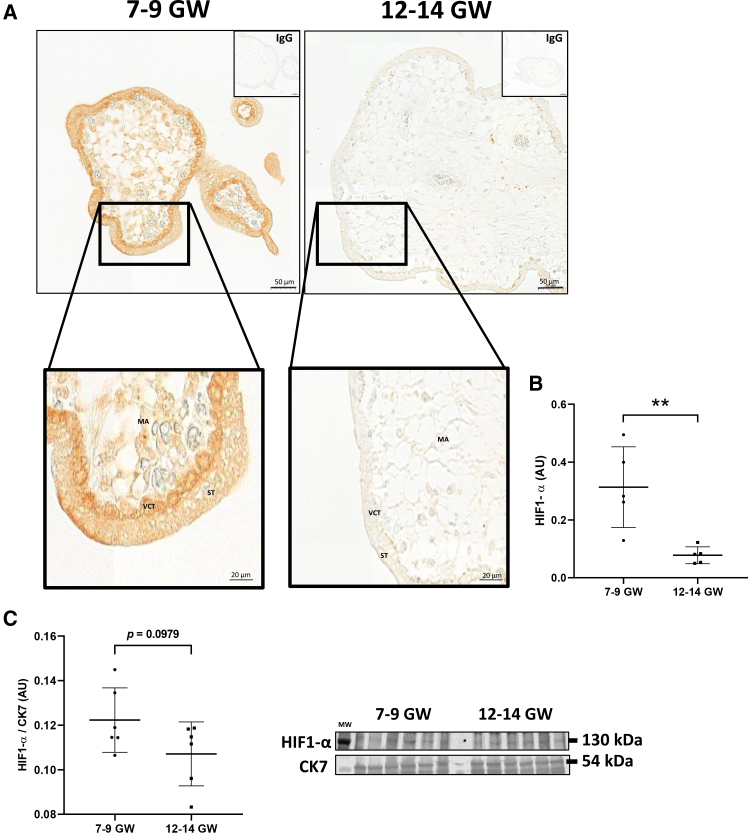
HIF1-α expression on early pregnancy (A) Chorionic villi are fixed in 4% PFA and included in paraffin. Immunostaining of HIF1-α is revealed with biotin-streptavidin-DAB system. Non-specific IgG staining was used as negative control (shown in the small box at the top right of each image). Scale bars, 50 μm. Magnified views of term chorionic villi are shown at the bottom of the panel. Scale bars, 20 μm. Experiments were reproduced 3 times per group. VCT, cytotrophoblast, ST, syncytiotrophoblast, MA, mesenchymal axis. (B) Quantification of HIF1-α immunostaining was performed from IHC images (*N* = 5 per group). Results are represented as mean (±) SD. Statistical analysis was performed using paired *t* test (∗∗*p* < 0.01). (C) Immunoblotting with an anti-HIF1-α antibody was performed on chorionic villi from 7 to 9 GW and 12–14 GW. CK7 was used as loading control. Graphs show the total amount of protein relative to CK7 level determined by quantification of immunoblot using ImageStudioLight from LICOR. Results are represented as mean (±) SD (*N* = 6 per group). Statistical analysis was performed using *t* test.

Immunolocalization of HIF1-α showed a strong marking in chorionic villi from 7 to 9 GW compared to 12–14 GW (Figure 1A), expression also quantified using Fiji software (Figure 1B) (*p* < 0.01). HIF1-α expression mainly localized in trophoblastic cells, at the periphery of chorionic villi, particularly in the cytoplasm of VCTs. Concerning protein expression of HIF1-α evaluated by western blot, immunoblots showed decreased expression after 12–14 GW, which is not statistically significant (*p* = 0.0979).

### Mitochondrial network is remodeled between 7–9 and 12–14 GW

**Figure 2:**
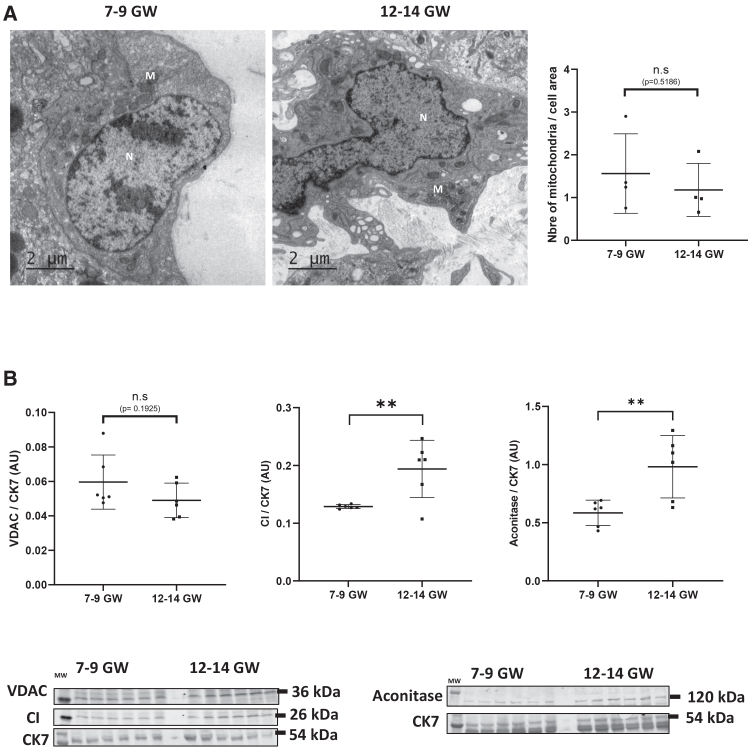
Remodeling of the mitochondrial network during early pregnancy (A) Chorionic villi from 7 to 9 GW and 12–14 GW were fixed and observed using TEM. Experiments are repeated three times per condition. Scale bars, 2 μm. Legend: N, nucleus, M, mitochondria. On the right, mitochondrial counts were performed based on the perinuclear surface area analyzed. (B) Immunoblot using antibodies against VDAC, complex I (CI) and aconitase was performed on chorionic villi from 7 to 9 GW and 12–14 GW. CK7 was used as a loading control. Graphs represent the total amount of protein relative to CK7 level determined by quantification of immunoblot using ImageStudioLight from LICOR. Results are represented as mean (±) SD (*N* = 6 for each group). Statistical analysis was performed using Mann-Whitney and *t* test depending on the distribution of the data. ∗*p* < 0.05, ∗∗*p* < 0.01.

Mitochondrial network in trophoblasts from first trimester chorionic villi was analyzed by TEM (Figure 2A) and mitochondrial protein levels were normalized to CK7 after verifying that CK7 expression was stable between the 7–9 and 12–14 GW groups. (Figures 2B and S1). TEM showed a dense perinuclear mitochondrial network in VCTs, both in chorionic villi from 7 to 9 GW and 12–14 GW (Figure 2A). Quantifying the number of mitochondria compared to perinuclear area showed no differences between both groups of chorionic villi (1.56 and 1.18 mitochondria/cell area at 7–9 and 12–14 GW) (Figure 2A). In this context, voltage-dependent anion channel (VDAC) protein level—an ubiquitous porin of the mitochondrial outer membrane—is also unchanged between 7 and 9 GW and 12–14 GW, indicating a stable number of mitochondria after villi chamber oxygenation. Nevertheless, protein levels implicated in mitochondrial metabolism are overexpressed in VCTs and ST from 12 to 14 GW compared to those of 7–9 GW. Indeed, proteins of both mitochondrial respiratory chain (complex I, CI) and Krebs cycle (*m*-aconitase) are significantly increased at 12–14 GW compared to 7–9 GW, from 0.13 to 0.2 and from 0.5 to 1 for CI and *m*-aconitase (*p* < 0.01), respectively.

### Mitochondrial fusion is induced at 12–14 GW

**Figure 3:**
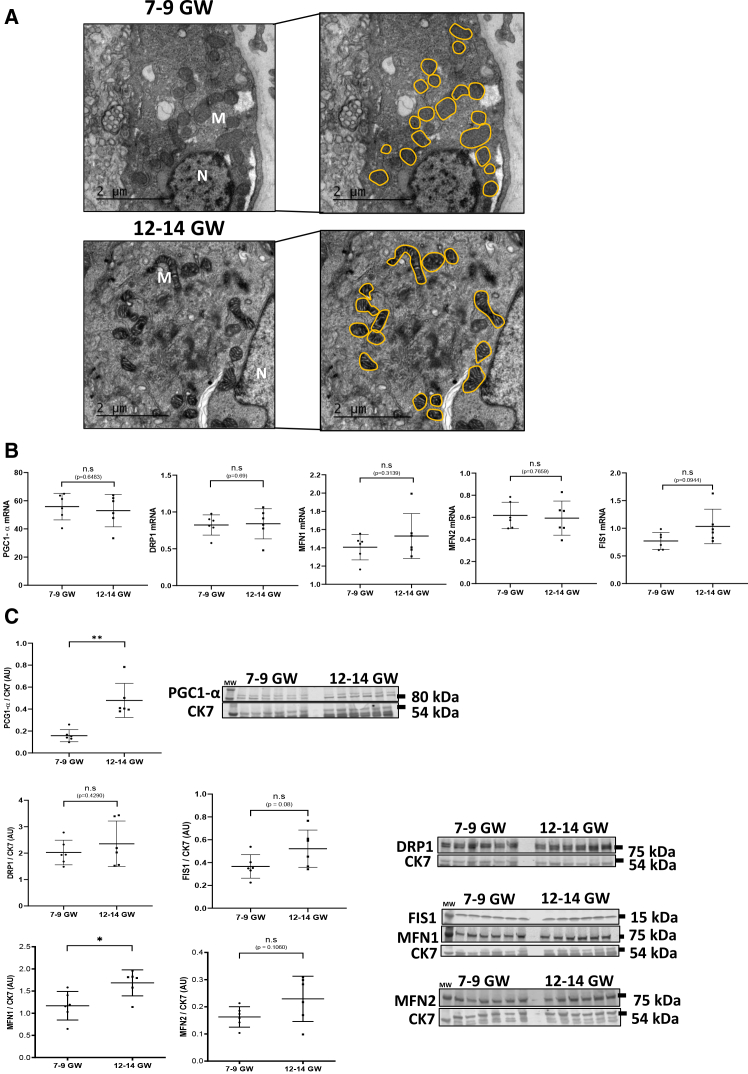
Mitochondrial dynamic during early pregnancy (A) Chorionic villi from 7 to 9 GW and 12–14 GW were fixed and observed using TEM, and mitochondria were outlined in orange. Experiments are repeated three times per condition. Scale bars, 2 and 5 μm. Legend: N, nucleus, M, mitochondria. (B) Level of PGC1-α, DRP1, FIS1, MFN1, and MFN2 in chorionic villi from 7 to 9 GW and 12–14 GW, measured by qPCR, normalized to the geometric mean of SDHA, RPLPO, and RPL13 transcripts. Statistical analysis is performed using paired *t* test (*N* = 6). Results are represented as mean ± SD. (C) Immunoblot using antibodies against PGC1-α, DRP1, FIS1, MFN1, and MFN2 antibodies was performed on chorionic villi from 7 to 9 GW and 12–14 GW. CK7 was used as loading control. The loading control image was reused (compared to Figure 2B); it is indeed the same membrane of western-blot. Graphs represent the total amount of protein relative to CK7 level determined by quantification of immunoblot using ImageStudioLight from LICOR. Results are represented as mean (±) SD (*N* = 6 for each group). Statistical analysis was performed using Mann-Whitney and *t* test depending on the distribution of the data. ∗*p* < 0.05.

Mitochondrial metabolism seemed increased at 12–14 GW without altering the number of mitochondria, so we thus questioned the mitochondrial morphology between 7–9 and 12–14 GW, and we especially studied the levels of proteins implicated in mitochondrial dynamic (Figures 3A and 3B). TEM images showed perinuclear, spherical mitochondria in ST and VCTs at 7–9 GW, while perinuclear mitochondria appeared more electron-dense with visible and large cristae at 12–14 GW (Figure 3A). Moreover, they surprisingly appeared particularly elongated at 12–14 GW compared to mitochondria from 7 to 9 GW (Figure 3A). This morphological shift was associated with a tendency for an increase in the level of proteins implicated in OMM dynamics, without significant changes at the transcript levels. Specifically, Pparg coactivator 1 alpha (PGC1-α), DRP1, MFN1, and MFN2 transcripts remained unchanged between 7 and 9 GW and 12–14 GW (Figure 3B). In contrast, protein expression of key regulators of mitochondrial fusion increased at 12–14 GW: MFN1 showed approximately a 50% increase (*p* < 0.05) and MFN2 exhibited a non-significant trend toward 30% (*p* = 0.1) (Figure 3C). FIS1 also displayed a trend toward higher transcriptional and protein levels at 12–14 GW (+20%) compared to 7–9 GW (Figures 3B and 3C), although this did not reach statistical significance. Finally, protein expression of PGC1-α—a key regulator of energy metabolism—is 3-fold higher at 12–14 GW compared to 7–9 GW (*p* < 0.01) (Figure 3C).

### Stabilization of HIF1-α by CoCl_2_ modulates the trophoblastic phenotype

**Figure 4:**
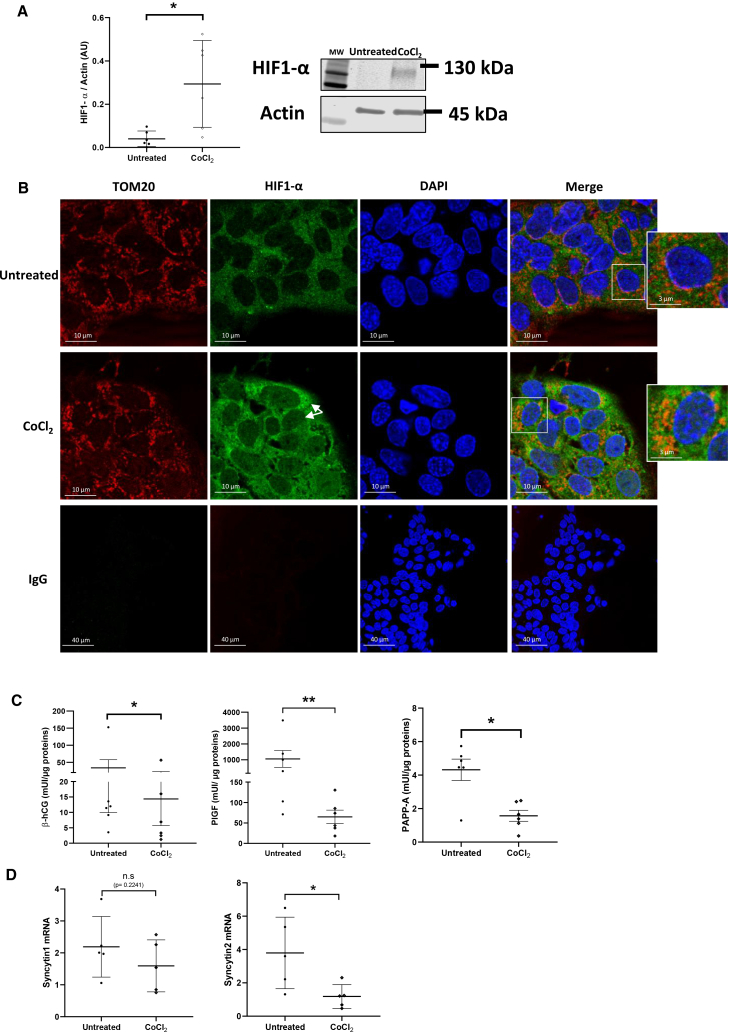
Trophoblastic phenotype after CoCl_2_ treatment (A) Immunoblot using HIF1-α antibody was performed on VCT exposed 24 h to CoCl_2_ (200 μM). Actin was used as loading control. Graphs represent the total amount of protein relative to actin level determined by quantification of immunoblot using ImageStudioLight from LICOR. Results are represented as mean (±) SD (*N* = 6). Statistical analysis was performed using Wilcoxon and paired *t* test depending on conditions. ∗*p* < 0.05. (B) VCT are exposed 24 h to CoCl_2_ (200 μM). Cells are then fixed in 4% PFA. Immunostaining of TOM20 ans HIF1-α are revealed with Alexa Fluor 546 (red) and Alexa Fluor 488 (green), respectively. Nuclei are marked with DAPI (blue). Experiments are reproduced 3 times per condition. Scale bars, 10 μm. Non-specific IgG is performed as negative control. Scale bars, 40 μm. (C) Total hCG, PlGF, and PAPP-A secretion after 24 h exposure to CoCl_2_ (200 μM). Statistical analysis was performed using paired *t* test (*N* = 6). Results are represented as mean (±) SD. ∗*p* < 0.05, ∗∗*p* < 0.01. (D) Level of Syncytin1 and Syncytin2 in chorionic villi from 7 to 9 GW and 12–14 GW, measured by qPCR, normalized to the geometric mean of SDHA, RPLPO, and RPL13 transcripts. Statistical analysis is performed using paired *t* test (*N* = 5). Results are represented as mean ± SD. ∗*p* < 0.05.

Because mitochondrial dynamics are modulated by changes in oxygen pressure, as observed during the first trimester and PE, we investigated the potential role of HIF1-α in initiating this process. To this end, VCTs were treated with 200 μM CoCl_2_ for 24 h, a well-established HIF1-α stabilizer at non-toxic dose, as verified by metabolic WST1 (water-soluble tetrazolium salt 1) assay (Figure S2). As expected, western blot analysis revealed a significant increase in HIF1-α protein levels following CoCl_2_ treatment (*p* < 0.05) (Figure 4A). Consistently, immunocytochemistry showed strong HIF1-α staining in both the cytoplasm and nucleus of CoCl_2_-treated VCTs compared to untreated controls (Figure 4B). HIF1-α staining colocalized with nuclear DAPI (4′,6-diamidino-2-phenylindole) (blue) and partially overlapped with mitochondrial TOM20 staining (red) in the cytosol (Figure 4B). These observations indicate that CoCl_2_ stabilizes HIF1-α, which localizes not only to the nucleus, consistent with its transcriptional role, but also in the cytosol near mitochondria, suggesting potential involvement in mitochondrial dynamics, metabolism, or stress response. The endocrine function of trophoblastic cells was evaluated by measuring the secretion of hCG, PlGF, and PAPP-A in the culture supernatants. Following CoCl_2_ treatment, all three hormones were significantly reduced (Figure 4C): hCG decreased from 45 to 14 mUI/μg proteins (*p* < 0.05), PlGF from 1,000 to 50 mUI/μg proteins (*p* < 0.01), and PAPP-A from 5 to 1.5 mU/μg proteins (*p* < 0.05). Finally, VCT fusion into ST was impaired as indicated by a significant reduction in Syncytin2 transcriptional levels, a key regulator of the fusion process, showing a 3-fold decrease compared to untreated cells (*p* < 0.05). Whereas Syncytin1 expression remained unchanged (Figure 4D). Notably, Syncytin1 is predominantly expressed in VCTs, while Syncytin2 is strongly expressed in ST, suggesting that CoCl_2_ specifically inhibits ST formation, which is consistent with the observed decrease in hCG secretion, as hCG is mainly produced by the ST.

The reduction in hCG, PlGF, and PAPP-A secretion is consistent with clinical biomarkers of PE. PE is also characterized by ST dysfunction, leading to the release of syncytial knots into the maternal circulation. Altogether, these results indicate that CoCl_2_-induced HIF1-α stabilization is associated with a PE-like trophoblastic phenotype.

### Stabilized HIF1-α triggers mitochondrial fission after CoCl_2_ exposure

**Figure 5:**
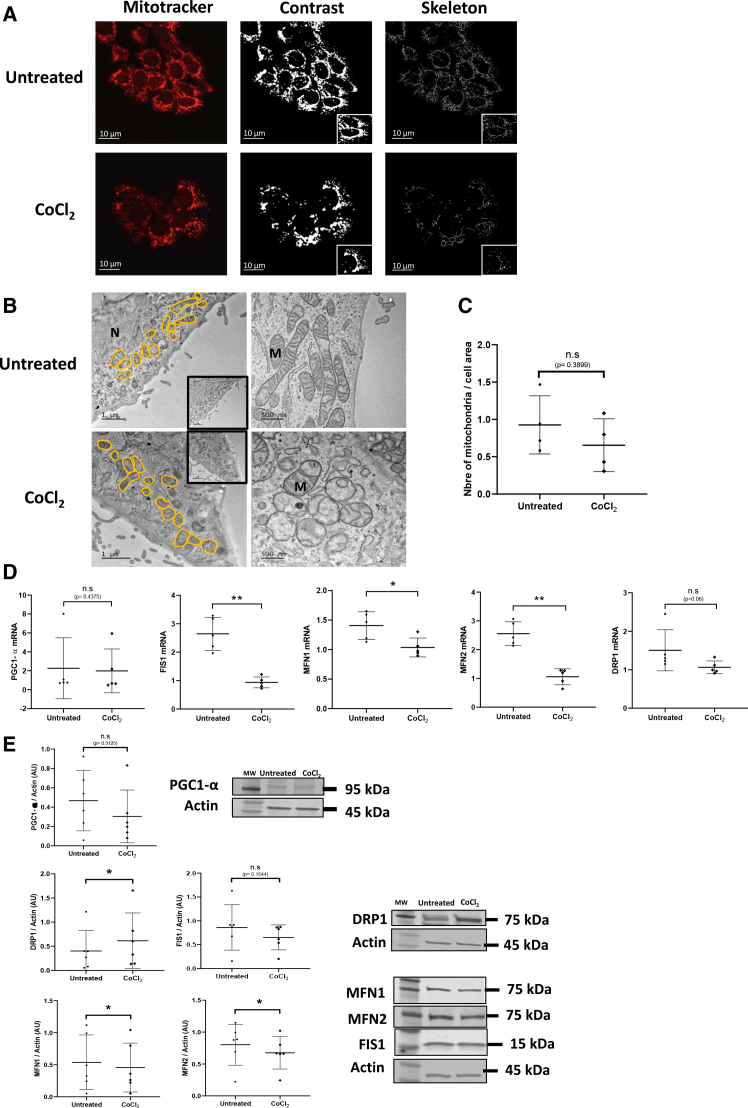
Mitochondrial dynamics after CoCl_2_ treatment (A) VCT were exposed to CoCl_2_ (200 μM) for 24 h and then exposed to CMX-ROS probe (50 nM). Cells were fixed in 4% PFA. Mitochondria was revealed with CMX-ROS probe (red). Mitochondria network was analyzed using ImageJ software. Experiments were repeated three times per condition. Scale bars, 10 μm. (B) VCT exposed 24 h to CoCl_2_ (200 μM) were fixed and observed by TEM. Mitochondria were contoured in orange. Scale bars, 1 μm and 500 nm. Legend: N, nucleus, M, mitochondria. (C) Mitochondria were counted based on perinuclear surface studied. Experiments were reproduced three times per condition. (D) Level of PGC1-α, DRP1, FIS1, MFN1, and MFN2 in chorionic villi from 7 to 9 GW and 12–14 GW, measured by qPCR, normalized to the geometric mean of SDHA, RPLPO, and RPL13 transcripts. Statistical analysis is performed using paired *t* test (*N* = 6). Results are represented as mean ± SD. ∗*p* < 0.05. (E) Immunoblot using PGC1-α, MFN1, MFN2, DRP1, and FIS1 antibodies was performed on VCT exposed 24 h to CoCl_2_ (200 μM). Actin was used as loading control. Graphs represent the total amount of protein relative to actin level determined by quantification of immunoblot using ImageStudioLight from LICOR. Results are represented as mean (±) SD (*N* = 6). Statistical analysis was performed using Wilcoxon and paired *t* test depending on the distribution of the data. ∗*p* < 0.05.

After 24 h of exposure of VCTs to CoCl_2_, mitochondrial network was first observed using the Mitotracker probe and analyzed using ImageJ software (Figure 5A). Mitochondria appeared mainly perinuclearly localized in both conditions. The network was less dense, more punctiform when VCTs were exposed to CoCl_2_; in particular, the mitochondrial skeleton was less reticulated and more fragmented (Figure 5A). Mitochondria were also observed by TEM that showed the same phenotype as observed with Mitotracker probe: mitochondria exposed to CoCl_2_ were small, rounded and a slightly reticulated inner membrane, suggesting mitochondrial fission processes (Figure 5B). Quantification of mitochondria per cell area showed no differences between untreated cells and those exposed to CoCl_2_ (Figure 5C). In addition, PGC1-α transcript and protein levels—a key regulator of metabolism and mitochondrial biogenesis—remained unchanged following CoCl_2_ exposure (Figures 5D and 5E). Concerning transcription levels of genes involved in mitochondrial fission: DRP1 level was unchanged after CoCl_2_ exposure while FIS1 expression was significantly reduced (around 2.5-fold, *p* < 0.05) (Figure 5D). Surprisingly, at the protein level, FIS1 was not significantly decreased while DRP1 level was increased by approximately 50% (*p* < 0.05) (Figure 5E). Concerning transcriptional levels of genes involved in mitochondrial fusion: CoCl_2_ induced a decrease in MFN1 (*p* < 0.05) and an even stronger reduction in MFN2 transcripts (*p* < 0.01) (Figure 5D). In line with this, at protein level, CoCl_2_ exposure significantly reduced MFN1 and MFN2 levels (decreasing them by 50%, *p* < 0.05) (Figure 5E). Altogether, western blot analysis confirms the shift toward mitochondrial fission process (increase of DRP1 and decrease of MFN1 and MFN2) in agreement with observations by confocal microscopy and TEM, suggesting that this remodeling process may be induced by HIF1-α stabilization. Interestingly, MFN1 and MFN2 protein and transcript levels were both decreased following HIF1-α stabilization, while the regulation of mitochondrial fission appeared more complex. Indeed, a clear discordance between transcriptional and protein levels was observed for DRP1 and FIS1, with opposite trends in their mRNA and protein expression.

### Enhanced mitochondrial fusion characterizes late PE

**Figure 6:**
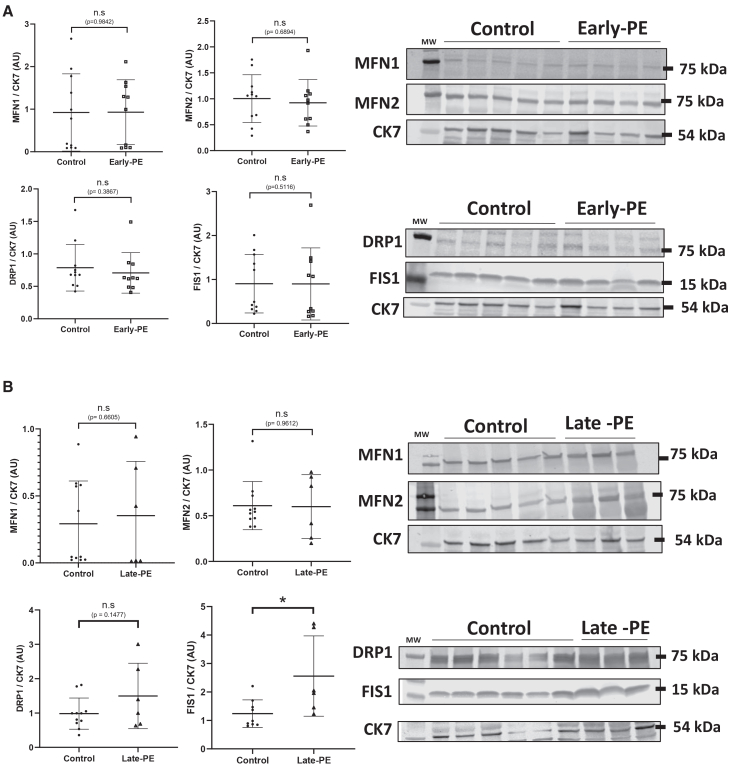
Mitochondrial dynamic during preeclampsia (A and B) Immunoblot using MFN1, MFN2, DRP1, and FIS1 antibodies was performed on chorionic villi from (A) early-preeclampsia (early-PE) and (B) late-preeclampsia (late-PE). CK7 is used as loading control. Graphs represent the total amount of protein relative to CK7 level determined by quantification of immunoblot using ImageStudioLight from LICOR. Results are represented as mean (±) SD. Statistical analysis was performed using Mann-Whitney and *t* test depending on conditions. ∗*p* < 0.05. *N* = 11 control, *N* = 10 early-preeclampsia and *N* = 6 late-preeclampsia.

Because mitochondrial dynamics are modulated during the first trimester in parallel with the physiological decline of HIF1-α levels, we investigated whether these dynamics were altered in PE, a condition associated with a hypoxic placental environment. According to our previous work, the cohort was constituted of two groups: early and late-preeclamptic women, compared to control women without any disease (Table 1). As previously performed on physiological placentas, mitochondrial proteins expression was normalized to CK7 level after verifying the stability of CK7 in both early- and late-preeclampsia (late-PE) (Figure S3). In early onset PE, no differences were observed in the expression levels of proteins implicated in mitochondrial dynamic (Figure 6A). In contrast, although late-PE is generally considered less severe than early-onset PE, expression of proteins implicated in mitochondrial fission was significantly increased: FIS1 level was 2-fold increased in late-PE compared to control, (*p* < 0.05) and DRP1 level tended to show a 1.5-fold increase (*p* = 0.1). These results thus suggest distinct mitochondrial phenotypes by type of PE, with high levels of proteins implicated in mitochondrial fission (DRP1 and FIS1).

### Results synthesis

**Figure 7:**
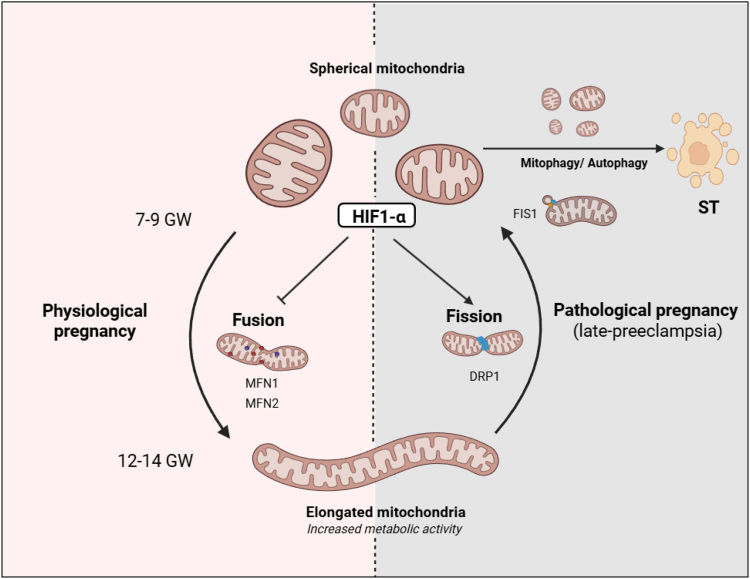
Mitochondrial dynamics on early pregnancy and during preeclampsia: implication of HIF1-α (using Biorender) GW, gestation week, ST, syncytiotrophoblast.

This work highlights a dichotomous balance between mitochondrial fusion and fission processes, which occur during early pregnancy—between 7 and 9 GW and 12−14 GW—and in late-PE (Figure 7). These physiopathological processes are closely associated with fluctuations in oxygen pressure. HIF1-α stabilization observed both during 7–9 GW and PE was associated with mitochondrial fission phenotype observed both by spherical and swollen mitochondria. In early physiological pregnancy, mitochondrial fission at 7–9 GW was linked to a significant downregulation of MFN1 compared to 12–14 GW, while during late-PE, mitochondrial fission was associated with a significantly increased expression of DRP1 and FIS1. *In vitro* stabilization of HIF1-α with CoCl_2_ suggests that HIF1-α promotes mitochondrial fission mechanisms by reducing MFN1 and MFN2 expression at both the transcriptional and protein levels, and by increasing DRP1 only at the protein level.

## Discussion

This study worked on the mitochondrial dynamics in physiological and pathological pregnancies, and researched the implication of HIF1-α—the key cell oxygen sensor—in this process. The rise of PaO_2_ from 10 GW, due to the removal of trophoblastic plugs in maternal uterine arteries induces as we have shown a drop in HIF1-α level in trophoblastic cells of chorionic villi.^3^ Oxygen transition was associated with changes in mitochondrial proteins implicated in metabolism. Complex I and *m*-aconitase levels were significantly increased after 12 GW, as is the mediator of mitochondrial metabolism, PGC1-α. Energetic balance is unchanged throughout the pregnancy, as shown by Jauniaux et al.,^21^ mentioning the constant ATP/ADP ratio in chorionic villi; nevertheless, metabolic needs of the feto-maternal unit are modified after the rise of PaO_2_ in the intervillous chamber.^22^ At poor PaO_2_, trophoblastic cells utilize metabolic pathways to handle carbohydrates (sorbitol, ribitol, and erythritol) that do not involve sugar phosphorylation.^21^ These pathways are interlinked to the pentose phosphate pathway and generate NAD+ (Nicotinamide adenine dinucleotide) and NADP+, which are used in glycolysis, requiring to support the high rate of cell proliferation, without developing dependence on lactate production.^21^^,^^23^ After PaO2 increase, oxidative phosphorylation is largely enhanced, inducing a higher Krebs cycle activity. These metabolic changes are thus modulated by oxygen availability but mostly by the nuclear protein PGC1-α. This last heterodimerizes with transcriptional factors, as peroxisome proliferator-activated receptor gamma (PPARγ), to activate transcription of genes implicated in glycolysis and oxidative phosphorylation.^24^ Depending on tissue and environmental needs, PGC1-α is also known as the initiator of mitochondrial biogenesis.^25^ In our work, oxygen transition and levels of mitochondrial proteins involved in cell metabolism were not associated with the increase of mitochondrial content between 7 and 9 GW and 12–14 GW. In parallel, HIF1-α stabilization with CoCl_2_ treatment showed no modulations of PGC1-α level and mitochondrial content. The increased expression of proteins implicated in metabolic pathways observed during the first trimester is thus dependent on oxygen presence (necessary for enzymatic activities) and the enhancement of PGC1-α, all this independently of HIF1-α level. These increased metabolic activities assure cell proliferation required for placental and fetus development. Furthermore, we observed an elongated mitochondrial morphology and large cristae after 12 GW, associated with a significantly increased protein expression of MFN1 (without affecting mRNA levels). MFN1 mediates the fusion of the OMM, involving a GTPase-driven reaction. Chen et al. have shown spherical mitochondria in mesenchymal cells from mouse placenta with MFN1 deletion (MFN1 −/−), leading to embryonic lethality at midgestation. The fusion of mitochondrial network is known to be positively associated with energy production (ATP synthase) and nutrient availability^26^; thus, hypertubulated mitochondria observed after 12 GW could reflect an increase in metabolic activity of highly proliferative trophoblasts.

HIF1-α is the main factor induced in low-oxygen environments, where it translocates to nucleus to activate target genes. Under normoxic conditions, two main mechanisms are involved in HIF1-α inactivation. First, the HIF inhibitory factor (FIH) is activated in the presence of oxygen and iron (Fe^2+^) and hydroxylate HIF1-α, preventing its interaction with the p300/CREB transcriptional coactivator complex. Specific prolyl hydroxylases (PHDs), which are also oxygen- and iron-dependent, hydroxylates HIF1-α, leading to its ubiquitination by the Von Hippel-Lindau protein (pVHL) and thereby targeting it for proteasomal degradation.^27^

To study the implication of hypoxia and more specifically HIF1-α in mitochondrial dynamics, we treated primary trophoblastic cells from term placentas with non-toxic dose of CoCl_2_ (Figure S2). This compound is known to chemically stabilize HIF1-α by inhibiting PHDs^27^ and is a commonly used alternative to hypoxia chambers. In addition, previous studies have shown strong similarities between low oxygen-induced hypoxia and CoCl_2_ in cellular metabolism (glycolytic gene activation)^28^ or signaling pathways activation (ROS production, mitogen-activated protein kinases [MAPK]).^29^ As previously mentioned, our work showed no modification in mitochondrial content and in the levels of proteins involved in mitochondrial metabolism after CoCl_2_ treatment (PGC1-α levels remained stable) but a significant change in mitochondrial phenotype was observed. Trophoblasts exposed to CoCl_2_ exhibited spherical perinuclear mitochondria that were poorly reticulated. At transcriptional level, CoCl_2_-induced HIF1-α stabilization led to a significant decrease of MFN1-2 and FIS1 (*p < 0.01*); three genes encoding proteins located at the mitochondrial outer membrane. At the protein level, CoCl_2_ also induced a significant decrease in MFN1and MFN2 (*p* < 0.05), in accordance with the transcriptional levels measured. Surprisingly, CoCl_2_ led to a significant increase in DRP1 protein expression (*p* < 0.05), while its transcriptional expression was not modified by CoCl_2_. Also, FIS1 protein levels appeared unchanged unlike mRNA. DRP1, ubiquitously expressed in cytosol, is recruited to mitochondria downstream of signaling cascades. It binds to mitochondrial adaptors at the surface of OMM, like FIS1, mitochondrial dynamics protein 49 or 51 (MiD49 or MiD51), and thanks to its retrograde transport via the dynein-dynactin complex, it constricts tubular membranes of mitochondria. DRP1 acts as the master regulator of fission; its silencing completely blocks fission process in mammalian cells and is thus sufficient for the severing of mitochondrial membranes.^14^^,^^26^ Our results suggest thus a regulation of mitochondrial dynamics by HIF1-α at two levels: transcriptional and post-translational. HIF1-α downregulated MFN1-2 genes transcription leading to a decrease of MFN1-2 protein levels and thus enhancing mitochondrial fission. Discrepancies between FIS and DRP1 mRNA and their protein levels revealed a potential interaction/regulation at post-translational levels. Mitochondrial fission is notably known to be regulated by post-translational phosphorylation on Serine585 or Serine616 of DRP1.^30^^,^^31^ HIF1-α, largely accumulated in cytosol after CoCl_2_ treatment, near mitochondria, could thus interact with DRP1 and induce an activating and stabilizing phosphorylation (Figure S4). Post-translational phosphorylation of DRP1 could also be indirectly enhanced by HIF1-α/MAPK or HIF1-α-Cdk1/cyclin B activation in proliferative trophoblasts,^30^ or by PINK1 (PTEN-induced putative kinase 1) activation in the context of mitophagy.^31^ All these observations led us to conclude that stabilization of HIF1-α, chemically enhanced by CoCl_2_ exposure, might be involved in mitochondrial fission in trophoblastic cells. These results are consistent with previous studies demonstrating mitochondrial fission induced by DRP1 after HIF1-α stabilization in various human cell types as human glioblastoma cells^32^ or human pancreatic beta cells.^33^

CoCl_2_-induced HIF1-α stabilization on primary VCT led to mitochondrial fission; as also observed in trophoblasts from 7 to 9 GW chorionic villi. For both tissues, fission was induced by a decrease in MFN1-2 protein levels (MFN1: *p* < 0.05 in both models and MFN2: *p* < 0.05 *in vitro* and *p* = 0.1 *ex vivo*). MFN1 mRNA levels were unchanged in 7–9 GW chorionic villi, indicating that the observed increase in MFN1 protein may result from an extended protein half-life. Such stabilization could be driven by post-translational mechanisms, including deacetylation or reduced ubiquitination of MFN1.^34^^,^^35^ Surprisingly, although in a context of mitochondrial fission, FIS1 protein tended to be reduced (without reaching significance) in trophoblast exposed to CoCl_2_ and in chorionic villi from 12 to 14 GW. It was associated with a stable expression of DRP1 in 7–9 GW chorionic villi, and an increased expression of DRP1 in VCT exposed to CoCl_2_. These notable results suggest that DRP1 is the master regulator of the fission process and is essential for inducing it while FIS1 appears only as a modulator of the process, depending on DRP1 expression. This hypothesis is supported by a recent study published in *Nature* showing that a knockout (KO) of DRP1 abolished all mitochondrial fission processes in Cos-7 cells while a KO of FIS1 only abolished a minor proportion of fission located at the periphery of mitochondria.^36^ These findings underscore that mitochondrial dynamics are predominantly driven by DRP1-dependent mechanisms, while FIS1 contributes only to specific, localized fission processes. The differences in mitochondrial dynamic mRNA and protein levels between *in vitro* and *ex vivo* models could be explained by the more complex structure of chorionic villi compared to primary culture of VCT. Chorionic villi are, indeed, composed of multiple placental cell types (mesenchymal, endothelial and immune cells) that can modulate, in a paracrine manner, cellular pathways and VCT/ST phenotype. Also, the gender of the first trimester chorionic villi has not been determined; recent studies suggest slightly different mitochondrial metabolism and biogenesis between male and female that could be interesting to evaluate.^37^ Furthermore, due to the villous structure, the cells of the chorionic villi are not uniformly exposed to oxygen (cells deeper within the tissue are less exposed); HIF1-α could therefore not be stabilized equally in all cell types, differentially modulating mitochondrial dynamics in chorionic villi.

Our results provide initial data concerning mitochondrial dynamics and HIF1-α implication in human placenta. Primary culture provides robustness of our results (interindividual variabilities considered), nevertheless it is technically very demanding and purified trophoblasts are relatively fragile (senescence after 96 h of culture). It is thus complex to explore in-depth signaling pathways using advanced models as KO or CRISPR-Cas9 that would be very useful to better understand fission process induced by HIF1-α. Although exposure to CoCl_2_ induces chemical hypoxia without affecting cell viability (Figure S2) and activates molecular and cellular pathways similar to those induced by atmospheric hypoxia,^27^ its action is not completely specific to HIF1-α. Acting on PHD activity, CoCl_2_ can also stabilize HIF2-α partner and may enhance ROS production, notably via Fenton reaction. This work thus establishes a correlation between HIF1-α stabilization and mitochondrial fission in trophoblastic cells of chorionic villi but doesn’t establish a direct link between these processes. HIF1-α is a key transcriptional factor in human cells; exploring its function during pregnancy is thus challenging because of the lethality of HIF1α^−/−^ cells. Analyzing mitochondrial phenotype after HIF1-α small interfering RNA (siRNA) knockdown on trophoblast cell lines (BeWo b30) would nevertheless provide valuable insights in future studies. The use of hypoxia chamber would also serve as an alternative method to stabilize HIF1-α and compare mitochondrial phenotypes. It should be noted that hypoxia chambers are technically challenging to use, and opening them can induce hypoxia-reperfusion events,^27^ which may alter the mitochondrial phenotype through cytosolic ROS generated by xanthine oxidase.^38^^,^^39^

To substantiate the consequences of hypoxia on mitochondrial morphology, we evaluated mitochondrial dynamics in cases of PE, a pregnancy disorder characterized by insufficient placental vascularization leading to hypoxia-reperfusion episodes.^40^ The cohort of preeclamptic patients was divided into early- and late-PE. The former is recognized as the earliest and most severe form of PE while the latter develops in the second half of the third trimester in predisposed women (e.g., obesity, cardiovascular diseases etc.).^40^^,^^41^ Our work showed no differences in mitochondrial dynamics-related proteins in early-onset PE while a significant increased expression of FIS1 (*p* < 0.05) was observed during late-PE, associated to a tendency increase of DRP1 (*p* = 0.1)., These results showed mitochondrial fission during late-PE as recently described by Gillmore et al. on mesenchymal cells from preeclamptic placentas.^42^ We thus observed distinct responses to mitochondrial dynamics between late and early-PE. The lack of modulations in fission proteins seen during early-PE could be explained by the potential insensitivity of early-PE trophoblast to the HIF1-α pathway by PHD mutations as already mentioned.^43^ Unfortunately, we were not able to study HIF1-α expression in preeclamptic tissues due to the scarcity of biological samples and the extremely rapid degradation of the transcription factor during delivery, constituting a limitation to understanding the potential impact of HIF1-α. Nevertheless, FIS1 overexpression in late-PE is not linked to HIF1-α stabilization based on our results obtained on chorionic villi at 7–9 GW and trophoblast exposed to CoCl_2_, in which FIS1 mRNA and protein levels showed a decreasing trend. A number of studies have shown that FIS1 is a key component of mitophagy induction, notably during hypoxia.^44^^,^^45^ Also, in the male germline of a mouse model, disruption of FIS1 leads to an increase of aberrant mitochondrial content and accumulation of mitophagic intermediates, suggesting defects in mitophagy.^46^ The recent study published by Pedrazzini and Manley, evaluated distinct fission processes associated with mitochondrial biogenesis or degradation on Cos-7 cells.^36^ This comprehensive study demonstrated at the molecular level that, DRP1 is involved both in midzone and peripheric fissions of mitochondria. Midzone fission generates two healthy daughter organelles, and thus contributes to mitochondrial biogenesis. FIS1 is predominantly expressed at the periphery of mitochondria, generating small daughter mitochondria from peripheral divisions that are directed toward degradation by autophagy or by lysosomes. In a mouse fibroblast cell line, the KO of proteins involved in fission-DRP1, mitochondrial fission factor (Mff), Mid49, Mid 51 of FIS1 (Drp1^−/−^, Mff−/−, Fis1^−/−^, Mid49−/−Mid51^−/−^)—showed that only FIS1 and Mff localize specifically at the periphery of mitochondria.^36^ Peripheral fissions are strongly enhanced after mitochondria-RE or mitochondria-lysosomes/phagosomes interactions suggesting that FIS1 could be upregulated by various signals of cell stress (lysosomal degradation and endoplasmic reticulum [ER] stress).^36^ In that sense, FIS1 protein is overexpressed in various pathologies (Parkinson’s disease, nephropathy, and diabetes)^47^ and recent studies suggest that FIS1 could be used as a biomarker of mitochondrial stress and mitochondria-related conditions.^48^ It would therefore be interesting to investigate the role of FIS1 in the human placenta, in particular its potential as a biomarker of late-PE.

To conclude, our work showed two distinct mechanisms leading to spherical mitochondria in human trophoblastic cells, depending on physiological or pathological context. During early physiological pregnancy (before 12 GW), spherical mitochondria are induced by the decreased expression of MFN1-2 in response to high levels of HIF1-α and consequently a reduced mitochondrial fusion. In case of preeclamptic pregnancy, the fragmented phenotype of the mitochondrial network is directly linked to the induction of the fission process by the increased expression of FIS1 and at lower extent, DRP1, in case of cellular stress. These two pathways might depend of the environmental oxygen accommodation and HIF1-α stabilization, but also the cellular needs and damages. Inhibition of mitochondrial fusion could be induced in physiological conditions by lower PaO_2_ to enhance mitochondrial metabolism and decrease steroid secretion, as also observed in VCTs compared to ST^10^ while, fission induction could result in mitochondrial damage, notably leading to mitophagy in late-PE.^42^^,^^49^^,^^50^^,^^51^ To explore the impact of HIF1-α stabilization/mitochondrial fission on trophoblast phenotype, we assessed hormones secretion and the fusion process after CoCl_2_ exposure to VCT. HIF1-α stabilization led to a decrease of three main placental hormones secreted by the ST, essential for the maintenance of pregnancy (hCG and PAPP-A) and placental angiogenesis (PlGF).^2^ Endocrine alteration was associated with a significantly reduced expression of syncytin-2, which is highly expressed in the ST and enables the fusion with the layer of VCT below.^52^ Trophoblasts exposed to CoCl_2_ have thus developed a phenotype similar to those of 7–9 GW (chorionic villi are mainly composed of proliferative VCT before 9 GW), supporting the impact of HIF1-α stabilization on trophoblastic phenotype. It now remains to be determined whether dysregulation of mitochondrial dynamics, through already known metabolic and steroid secretion consequences,^10^^,^^11^ may contribute to or exacerbate the development of placental pathologies such as PE, intrauterine growth restriction and gestational diabetes.^53^

### Limitations of the study

Our results provide initial data concerning mitochondrial dynamics and the involvement of HIF1-α implication in the human placenta. As mentioned in the discussion Section, although exposure to CoCl_2_ induces chemical hypoxia and activates molecular and cellular pathways similar to those triggered by atmospheric hypoxia,^27^ its action is not completely specific to HIF1-α. This work therefore establishes a correlation between HIF1-α stabilization and mitochondrial fission in trophoblastic cells of chorionic villi but does not establish a direct causal link between these processes. Due to the fragility of primary trophoblasts, it was not possible to refine our understanding of molecular mechanisms by which HIF1-α regulates mitochondrial dynamics, particularly through the use of siRNA; a model that could however be applied trophoblastic cell lines (BeWo b30).

## Resource availability

### Lead contact

Requests for further information and resources should be directed to and will be fulfilled by the lead contact, Amal Zerrad-Saadi (amal.zerrad-saadi@u-paris.fr).

### Materials availability

This study did not generate new unique reagents.

### Data and code availability

• All data reported in this paper will be shared by the lead contact upon request.
• This paper does not report original code.
• Any additional information required to reanalyze the data reported in this paper is available from the lead contact upon request.

## Acknowledgments

This research was supported by the 10.13039/501100001677Institut National de la Santé et de la Recherche Médicale (INSERM) and Université de Paris Cité. The authors would like to thank the platform “PICMO,” Faculty of Pharmacy of Paris, Paris Cité University, France, for access to a Leica confocal microscope, in particular B. Saubamea for TEM (Jeol) tissue observations, and the histology, immunostaining, and laser microdissection “HistIM” Department, Institut Cochin, 27 rue du Faubourg Saint-Jacques, 75014 Paris for access to the slide scanner LAMINA. The following are acknowledged for providing placentas: Hôpital Cochin-Port Royal, maternité privée des Diaconesses, Institut Mutualiste Montsouris, Hôpital privé d’Antony, and CHU de Lille.

## Author contributions

L.P. performed experiments and drafted the manuscript; A.C. and J.C. performed western blotting and qPCR; B.L. performed biochemical assays; C.I. realized electronic microscopy observations; L.B., J.-L. B., and T.F. revised the manuscript; I.F. and I.H. participated in the development of the project and revised the manuscript; A.Z.-S. wrote, supervised, and revised the manuscript. All authors read and approved the final manuscript.

## Declaration of interests

The authors declare no competing interests.

## STAR★Methods

### Key resources table

**Table:** 

REAGENT or RESOURCE	SOURCE	IDENTIFIER
**Antibodies**		

Monoclonal anti-DRP1	Novus	Cat# 16232
Polyclonal anti-FIS1	Sigma	Cat# HPA017430
Monoclonal anti-MFN1	Cell Signaling	Cat# 14739
Monoclonal anti-MFN2	Abcam	AB_56889
Polyclonal anti-HIF1-α	Novus	AB_ NB100-449
Polyclonal anti-PGC1-α	Abcam	Cat# 54481
Polyclonal anti-OPA1	Abcam	Cat# 42364
Anti-complex I (anti-NDUFS3)	Mitoscience	Cat# MS112
Anti-VDAC	Homemade: Pr Catherine Brenner	N/A
Anti-m-aconitase	Homemade: Dr R.B Franklin	N/A
Monoclonal anti-CK7	Dako	Cat# M7018

**Biological samples**		

Term placentas (37-39 GW)	Volunteers	N/A
1^st^ trimester placentas (7-14 GW)	Volunteers	N/A
Preeclamptic placentas	Volunteers	N/A

**Chemicals, peptides, and recombinant proteins**		

Trypsin	Gibco	#27250-018
DNase type 4	Sigma	#SLBV9316
Percoll^TM^	Cytiva	#17544501
Cobalt Chloride	Sigma	#255599

**Critical commercial assays**		

Orange CM-H2TMRos	ThermoFisher	#M7512
ARNPicoPure^TM^	FisherScientific	#KIT0204
PowerTrackSYBR Green^TM^ Master Mix	FisherScientific	#10459604
WST1 assay	Roche	#11644807001

**Experimental models:cell lines**		

Primary culture of trophoblast	Purification from human placenta	N/A

**Oligonucleotides**		

Primers against DRP1 5’-GGCAACTGGAGAGGAATG-3’ 5’- GGCATGACCTTTTTGTGG-3’	Eurogentec	N/A
Primers against FIS1 5’-ACCTGGCCGTGGGGAACTACC-3’ 5’-AGTTCCTTGGCCTGGTTGTTCTGG-3’	Eurogentec	N/A
Primers against MFN1 5’- AGGTGGCCGTGGGGAACTACC-3’ 5’-CCAAAACACACGTACAAGACAG-3’	Eurogentec	N/A
Primers against MFN2 5’-ATCCCCACTTAAGCACTTTGTC-3’ 5’-ATTCCTGTACGTGTCTTCAAGG-3’	Eurogentec	N/A
Primers against PGC1-α 5’-ACCACTCCTCCTCATAAAGCC-3’ 5’- TGCCTTGTGTACCAGAAGACTC-3’	Eurogentec	N/A
Primers against RPL13 5’-CTGGCGTGGAAATAGGTGAT-3’ 5’-CGGCCCACCTTAACTTTACA-3’	Eurogentec	N/A
Primers against RPLO 5’-TCGACAATGGCAGCATCTAC-3’ 5’-GCCTTGACCTTTTCAGCAAG-3’	Eurogentec	N/A
Primers against SDHA 5’-TGGGAACAACAGGGCATCTG-3’ 5-CCACCACTGCATCAAATTCATG-3’	Eurogentec	N/A
Primers against Syncytin1 5’-CGGACATCCAAAGTGATACATCCT-3’ 5’-TGATGTATCCAAGACTCCACTCCA-3’	Eurogentec	N/A
Primers against Syncytin2 5’-GCCTGCAAATAGTCTTCTTT-3’ 5’-ATAGGGCTATTCCCATTAG-3’	Eurogentec	N/A

**Software and algorithms**		

Prism 9	GraphPad	graphpad.com
Fiji ImageJ v1.51	Fiji	imagej.net
ImageStudio_6.0.0.28	Licorbio	www.licorbio.com
SlideViewer	3DHistech	www.3dhistech.com

### Experimental model and study participant details

#### Ethics statement

The study conformed to the Declaration of Helsinki. Placental tissues (first trimester and term placenta) used for this study were obtained after the patients’ written informed consent from Cochin Port-Royal maternity unit, Anthony Hospital, Diaconesses maternity, Mutualist Institute, and Lille Hospital. The protocol was approved by the institutional review board (Comité de Protection des Personnes 2015-mai-13909). Prior to taking part to the study, patients provided their written consent and personal details were anonymized through assignment of unique collection number.

#### Placenta retrieval from early pregnancy

Following French law, placentas were obtained from singleton pregnancies after voluntary selective terminations of pregnancy (first trimester, 7-9 GW/12-14 GW). The placentas provided by an assisted reproduction procedure, miscarriages, or in a context of fetal abnormalities or genetic diseases were not included.

After collection of all the chorionic villi from the placentas, they were washed in HBSS at 37°C and dissected free of membranes. The tissues were quickly frozen and stored at -80°C, or fixed in 4 % paraformaldehyde and embedded in paraffin for immunohistochemistry, or fixed in 1.5 % paraformaldehyde (PFA)- 1.5 % glutaraldehyde (GA) for transmission electron microscopy. The sex of the placentas collected is unknown due to their early collection (first trimester of pregnancy). The limitations encountered are reported in the paper, in the discussion section.

#### Preeclamptic tissus collection

PE was defined by a systolic pressure ≥ 140 mmHg or a diastolic pressure ≤ 90 mmHg, associated with proteinuria greater than 300 mg/24 h, after 20 GW, or a P/C ratio ≥ 30 mg/mmol. Preeclampsia (PE) was divided into two groups: early-onset PE (< 34 GW) and late-onset PE (> 34 GW).

This study used the same cohort of preeclamptic patients as our previously published study.^7^ It is composed of 17 preeclamptic women divided into two groups: early-onset PE (Early-PE < 34 GW, N = 11) and late-onset PE (Late-PE > 34 GW, N = 6). A control group was constituted of 10 healthy pregnant control women (delivery at term: 37-39 GW). Clinical details are summarized in Table 1. Women with HELLP-syndrome were excluded and all groups were age-matched. As expected, the Early-PE group had a lower gestational age (*p* < 0.0001) and birthweight (*p* = 0.0002) than the control group while no differences were observed between the Late-PE and control groups. Maternal age and placental sex were not statistically different between groups.

**Table 1:** Characteristics of patients and neonatal outcomes

	1 Control (*N* = 10)	2 Early-PE (*N* = 11)	3 Late-PE (*N* = 7)	*p* values (1 versus 2)	*p* values (1 versus 3)	*p* values (2 versus 3)
**Characteristics of participants**						

Age (years)	**30.6** ± 4.8 (*N* = 5/11)	**29.1** ± 5.0 (*N* = 10/10)	**27.3** ± 6.3 (*N* = 6/6)	0.8690	0.5872	0.8040
Anterior gestation (nb)	**3.4** ± 1.7 (*N* = 5/11)	**1.4** ± 0.7 (*N* = 10/10)	**2.2** ± 1.5 (*N* = 6/6)	0.0443	0.6849	0.7218
Parity (nb)	**3.4** ± 1.7 (*N* = 5/11)	**1.4** ± 0.7 (*N* = 10/10)	**1.7** ± 1.4 (*N* = 6/6)	0.0641	0.2231	>0.9999

**Neonatal outcomes**						

Gestation age at delivery (wk)	**38.8** ± 1 (*N* = 11/11)	**30.1** ± 2.2 (*N* = 10/10)	**38** ± 1.1 (*N* = 6/6)	<0.0001	0.8995	0.0222
Birthweight (g)	2963 ± 1216 (*N* = 7/11)	**1164** ± 313 (*N* = 10/10)	**3113** ± 574 (*N* = 6/6)	0.0003	0.9320	0.0002
Gender (♀ = 1, ♂ = 0)	**0.33** ± 0.52 (*N* = 6/11)	**0.4** ± 0.52 (*N* = 10/10)	**0.33** ± 0.52 (*N* = 6/6)	>0.9999	>0.9999	>0.9999
Type of delivery	caesaraean (*N* = 11/11)	caesaraean (*N* = 10/10)	caesaraean (*N* = 6/6)	–	–	–

Results are represented as mean ± standard deviation. Statistical analysis was performed using ANOVA (normal distribution) and the Kruskal-Wallis test (since the data do not follow a normal distribution).

#### Villous cytotrophoblast (VCTs) purification

VCTs were isolated from human term placentas as previously described.^54^ The basal layer was gently taken off and pieces of cotyledon were cut and rinsed in Ca^2+^ and Mg^2+^-free HBSS. The dissection did not exceed 30 min to avoid tissue degradation. First, the chorionic villi were incubated without agitation at 37°C in digestion buffer containing 1 % trypsin (5 mL/g), 100 mmol/L MgSO_4_, 100 mmol/L CaCl_2_, 25 IU DNase, and 7 % semi-skimmed milk. After 30 min, chorionic villi were incubated 3-4 times in the trypsin solution for 10 min at 37°C until the dissociated cells appeared. Warm HBSS was used to wash the chorionic villi and supernatants containing a majority of VCTs were collected and pooled, filtered (40 μm pores), and incubated with 10 % fetal calf serum (FCS, v/v) to stop trypsin activity. Next, cell suspensions were centrifuged (1200 rpm for 10 min) at room temperature. Cell pellets were suspended in DMEM, layered onto a Percoll® gradient (60 %, 50 %, 45 %, 35 %, 30 %, 20 %, 10 %), and centrifuged at 2500 rpm at room temperature. After 20 min, the layer between the 45 % and 35 % of Percoll® containing trophoblastic cells was collected and resuspended in DMEM. Cells were then centrifuged at 1200 rpm for 10 min and the resulting cell pellet was re-suspended in complete DMEM (phenol red-free, 1 % glutamax, 1 % penicillin-streptomycin) supplemented with 10 % FCS. VCTs were counted using a TC20 automated cell counter from Biorad, seeded at a density of 125,000 cells/cm^2^ on 35-mm culture dishes and incubated overnight in 5 % CO_2_ at 37°C.

#### Cobalt chloride treatment

After 12 h of culture, cells were treated with 200 μM of CoCl_2_ for 24 h. After treatment, cells were used for protein extraction and the supernatants were collected for hCG, PlGF, and PAPP-A quantification.

### Method details

#### Immunohistochemistry

Placenta tissues (N = 3: first trimester) were fixed in 4 % paraformaldehyde for 4 h at 4°C, dehydrated and embedded in Paraplast® (Leica Biosystems, Fisher Scientific, Illkirch, France). Placental tissue sections of 5 μm were deparaffinated in Safe-Solv (Labonord, Templemars, France) and rehydrated in ethanol/water (v/v). Sections were permeabilized in 0.3 % Triton X-100/ PBS for 4 min, and then washed 3 times for 5 min with PBS. Antigens were then retrieved by treatment, for 40 min at 90°C, in citrate buffer, pH 6.1 (Dako, Trappes, France). Endogenous peroxidase activity was blocked with 3 % hydrogen peroxide for 30 minutes and non-specific binding was blocked with 7 % BSA and 12.5 μg/mL human IgG (Jackson ImmunoResearch Laboratories, Interchim, Montlucon, France), for 1 h at room temperature. Immunostaining was performed with a streptavidin–peroxidase immunostaining kit (LSABH+Kit DAKO, Trappes, France) using specific antibody against HIF1-α (2 μg/mL) or non-specific rabbit IgG as control. Tissue sections were mounted in Faramount aqueous mounting medium (Dako) and the slides were scanned on a Lamina multilabel scanner (Perkin Elmer).

#### Multiplex western blot analysis

Protein extraction from first trimester and term villi, and purified VCTs was carried out using RIPA buffer containing 0.01 % of a protease inhibitor cocktail and 0.01 % of a phosphatase inhibitor cocktail. Villi were homogenized with an Ultra-Turrax homogenizer, and VCTs were sonicated for 2 min and then centrifuged at 10,000 rpm for 10 min at 4°C. Supernatants were collected and stored at -80°C.

Protein concentrations were determined using the Micro BCA Protein Assay Kit following manufacturer’s instructions, and absorbance was measured using an Enspire® spectrophotometer (PerkinElmer).

After denaturation at 60°C for 1 h, protein extracts were separated by 10 % SDS-PAGE and immunoblotted with primary antibodies against MFN1, MFN2, DRP1, FIS1, HIF1-α, PGC1-α, VDAC, m-aconitase, Complex I (CI), and cytokeratin7 (CK7) (all at 1:1000). After incubation with Alexa Fluor-conjugated secondary antibodies (680 or 800; Molecular Probes, Fisher Scientific, Illkirch, France), blots were revealed by using an Odyssey® infrared imaging system and then analyzed with Li-COR Odyssey® software v3.0 (Li-Cor Bioscience, Lincoln, NE, USA) and ImageStudioLight.

#### Electronic microscopy

Chorionic villi and VCTs were fixed in a mix of 1.5 % glutaraldehyde and 1.5 % paraformaldehyde in sodium cacodylate pH 7.3 buffer, for 1 h at room temperature. After one night in sodium cacodylate buffer, a secondary fixation was performed with 1 % OsO_4_ and 1 % potassium ferrocyanide in 0.1 M sodium cacodylate buffer (1 h, 4°C). Then, tissues and cells were stained in 1 % uranyl acetate for 1 h at room temperature. They were then dehydrated in a graded ethanol series (30 %, 50 %, 70 %, 95 % and 100 %) followed by embedding in Epon epoxy resin. Ultrathin sections (80 nm thickness) were observed using a Jeol electron microscope (JEM-100S, Croisy sur Seine, France) at 80 kV with an Orius SC200 digital camera (Gatan-Roper Scientific, Evry, France). Mitochondrial counting in trophoblastic cells was performed using Fiji software. The count was reported relative to the measured cell surface area (arbitrary unit).

#### RT-q.PCR

Primers were designed so that amplification products ranged between 100 and 250 bp. Sequences of primers (already published (22)) are presented in key resources table. PicoPure^TM^ kit was used to extract total RNA from VCT. RNA was then quantified with a NanoDrop spectrophotometer and reverse-transcribed into cDNA (stored at -20°C). Reverse transcription was performed on 1 μg of RNA using the SuperScript III First Strand Synthesis System (Invitrogen #18080044, Villebon-sur-Yvette, France) and random primers (Invitrogen # 48190011). The quantitative real-time PCR was performed in a 10 μL reaction volume containing 100 ng of cDNA, 0.5 μM of each primer, and SYBR™ MasterMix (SYBR® MasterMix dTTP Blue, Eurogentec, Angers, France). Forty cycles were carried out using an ABI Prism 7900 Sequence Detection System (Applied Biosystems). Q.PCR was performed using the following steps: Taq polymerase activation (15 min, 95°C), denaturation (30 s, 94°C), annealing (30 s, 55°C), extension (30 s, 72°C), and final extension (10 min, 72°C). mRNA levels were normalized by the geometric averaging of housekeeping genes: SDHA, RPLP0 and RPL13. The Ct (threshold cycle) was measured as the number of cycles required for the fluorescent signal to cross the threshold (exceeded background level) and the relative amounts of mRNA were measured using the ΔCt method.

#### TOM20 and HIF1-α immunofluorescence staining

Trophoblastic cells were fixed in 4% PFA for 20 min at room temperature. Cells were washed in PBS 3 times and then treated with NH4Cl 50mM for 10 min. Permeabilization was performed in 3% PBS- 0.5% Triton for 10 min and then blocked for 1h at room temperature in a solution containing: 5% donkey serum, human IgG (12.5 μg mL-1) and 0.3% Triton. VCT were incubated overnight with specific antibodies against TOM20 (Mouse, 2 μg mL^-1^) and HIF1-α (Rabbit, 2 μg mL^-1^), in PBS-1% BSA – 0.1% triton. Non-specific IgG was used as a control. Cells were washed and incubated 1h, at room temperature, with AlexaFluor 488 (1/500, directed against HIF1-α) and AlexaFluor 546 (1/500, directed against TOM20). Nuclei were stained with DAPI (1/10 000, 5 min). Cells were observed using an SP8 confocal microscope (Leica).

#### MitoTracker CMXRos

Cells were washed with HBSS and treated with Mitotracker CM-H2TMRos following manufacturer’s instructions. CM-H2TMRos probe was diluted at 50 nM in complete culture medium. Cells were incubated for 30 min at 37°C with the probe, which was then removed and cells were washed in PBS. Fixation was performed using 4 % PFA, for 20 min at room temperature before adding TOPRO-3 (1:500) for 10 min. Slides were then finally mounted with fluorescent mounting medium and observed with a SP8 confocal microscope (Leica).

#### Viability assay (WST1)

Viability was measured using the WST-1 assay (Sigma-Aldrich #11644807001). It measures the metabolic activity of trophoblasts studying the cleavage of the tetrazolium salt 2-(4-iodophenyl)-3-(4- nitrophenyl)5-(2,4-disulfophenyl)-2H-tetrazolium (WST-1) into formazan by dehydrogenases. VCT were treated for 24 h with CoCl2 (200 μM). Cells were then rinsed with culture medium and WST-1 reagent was added (1:100). Following manufacturer instructions, after 3 h incubation at 37°C, absorbance was measured using a microplate reader (EnSpire 2300 Multilabel reader, PerkinElmer, Villebon-sur-Yvette, France) at 440 nm. Nonspecific emission at 600 nm was measured and omitted from the initial value.

#### hCG, PlGF and PAPP-A secretion assay

After centrifugation (200 g, 10 min) of culture supernatants from primary VCTs, assays were performed as follow: (a) for total hCG using a chemiluminescent microparticle immunoassay (CMIA). The analysis was performed with an Alinity reagent kit and analyzer (Abbott) in an accredited (Cofrac) clinical chemistry laboratory- (b) for PlGF using a commercial kit validated as a tool for screening of pre-eclampsia (DELFIA Xpress PlGF 1-2-3 assay, Perkin Elmer, two-site fluoroimmunometric assay) – (c) for PAPP-A using a commercial kit (DELFIA Xpress, Revvity) analyzed by Dalphia Xpress instrument.

### Quantification and statistical analysis

All data were analyzed with GraphPad Prism software (La Jolla, CA, USA). Normality was systematically evaluated with a Shapiro test and the variance equality by a Fisher test. When both conditions were validated, parametric t-test was practiced. In a case of non-parametric and unpaired data, Mann-Whitney test was applied (Wilcoxon test for paired data). A p-value lower than 0.05 was considered statistically significant, with *p* < 0.05, *p* < 0.01 or *p* < 0.001 represented as ∗, ∗∗ or ∗∗∗ respectively. Graphical representations show experimental results with mean ± SD and the number of biological replicates is indicated by ‘n =’.
