# Supplementary material for: HIF1-α induces mitochondrial fission in trophoblastic cells during early pregnancy and in preeclampsia
Source: iScience. 2026 Apr 9;29(5):115674. doi: 10.1016/j.isci.2026.115674 (PMC13138064; doi:10.1016/j.isci.2026.115674)
Supplement: Document S1. Figures S1–S4 [file mmc1.pdf]

iScience, Volume *29*

Supplemental information

HIF1-α induces mitochondrial fission in trophoblastic cells during early pregnancy and in preeclampsia

Léa Poinsignon, Audrey Chissey, Juliette Colombel, Bertrand Lefrère, Charlotte Izabelle, Lucie Bernard, Jean-Louis Beaudeux, Thierry Fournier, Ioana Ferecatu, Isabelle Hernandez, and Amal Zerrad-Saadi

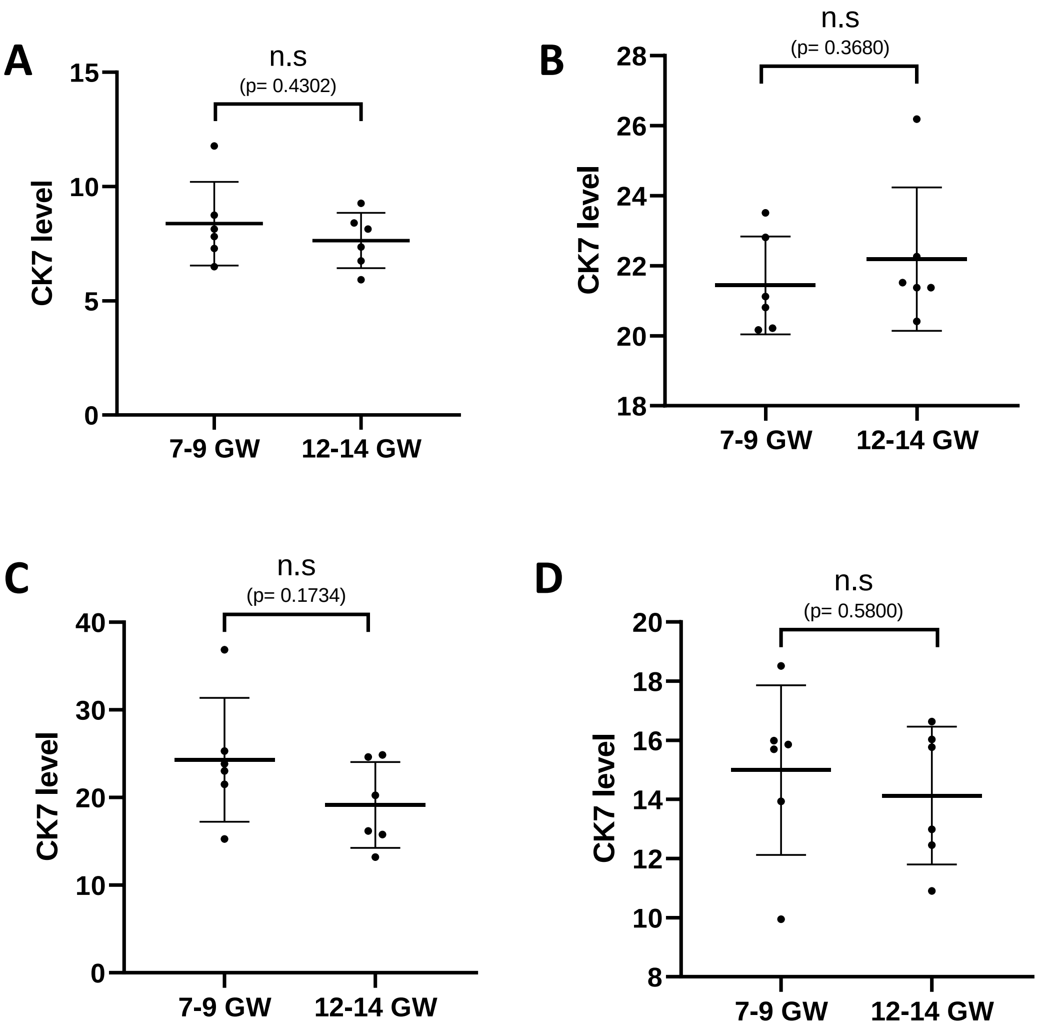

Figure S1: Cytokeratin 7 level between 7-9 GW and 12-14 GW

Level of CK7 in chorionic villi from 7-9 GW and 12-14 GW for Western-Blot membrane 1 (A), membrane 2 (B), membrane 3 (C) and membrane 4 (D). For each sample, 40 µg of total proteins were loaded on electrophoresis gels. Graphs represent the total amount of protein CK7 level determined by quantification of immunoblot using ImageStudiolight from LICOR. Results are represented as mean (±) SD (N = 6 for each group). Statistical analysis was performed using Mann-Whitney and t-test depending on conditions (n.s for non-significant).

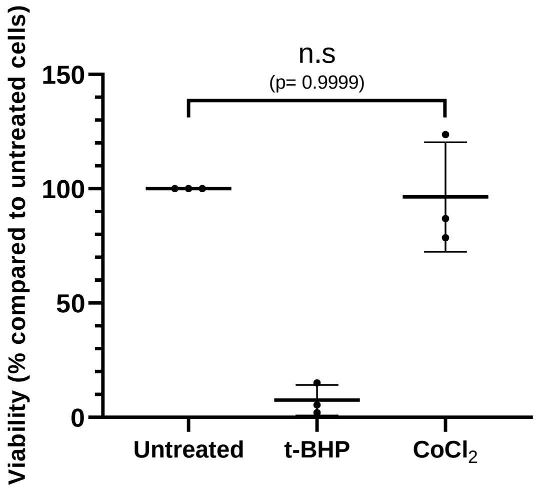

Figure S2: Viability of VCT treated with $CoCl_2$

VCT are exposed to $CoCl_2$ (200 µM) or t-BHP (200 µM) for 24 h in 48-well plate. VCT metabolic activity is measured by WST1 assay. Each condition is reported to control activity (VCT not exposed). Experiments are represented as mean (±) SD (N=3). Statistical analysis was performed using Wilcoxon-test.

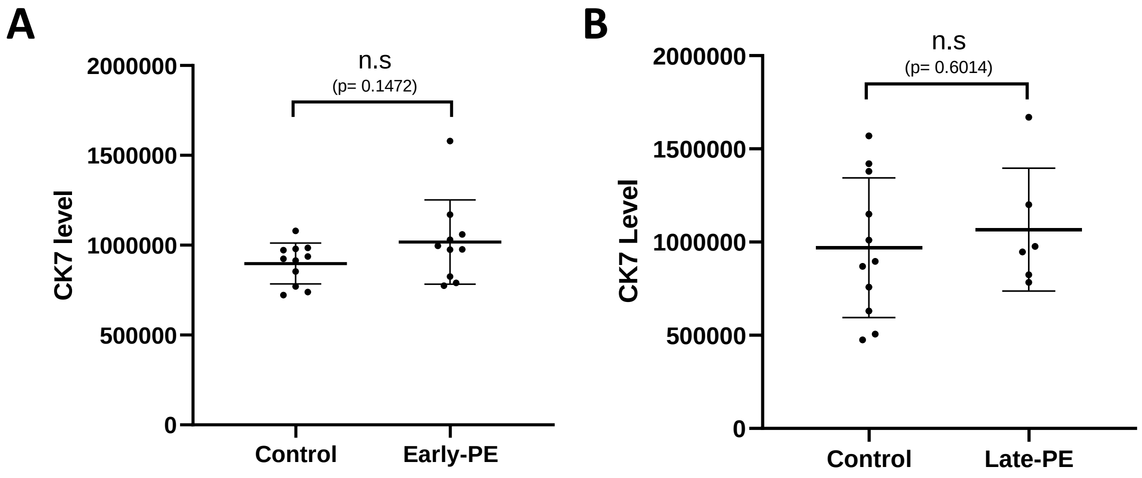

Figure S3: Cytokeratin 7 level between Early and Late -Preeclampsia

Level of CK7 in chorionic villi from Early- (A) and Late-PE (B) compared to control. For each sample, 40 µg of total proteins were loaded on electrophoresis gels. Graphs represent the total amount of protein CK7 level determined by quantification of immunoblot using ImageStudiolight from LICOR. Results are represented as mean (±) SD (N = 6 for each group). Statistical analysis was performed using t-test (n.s for non-significant).

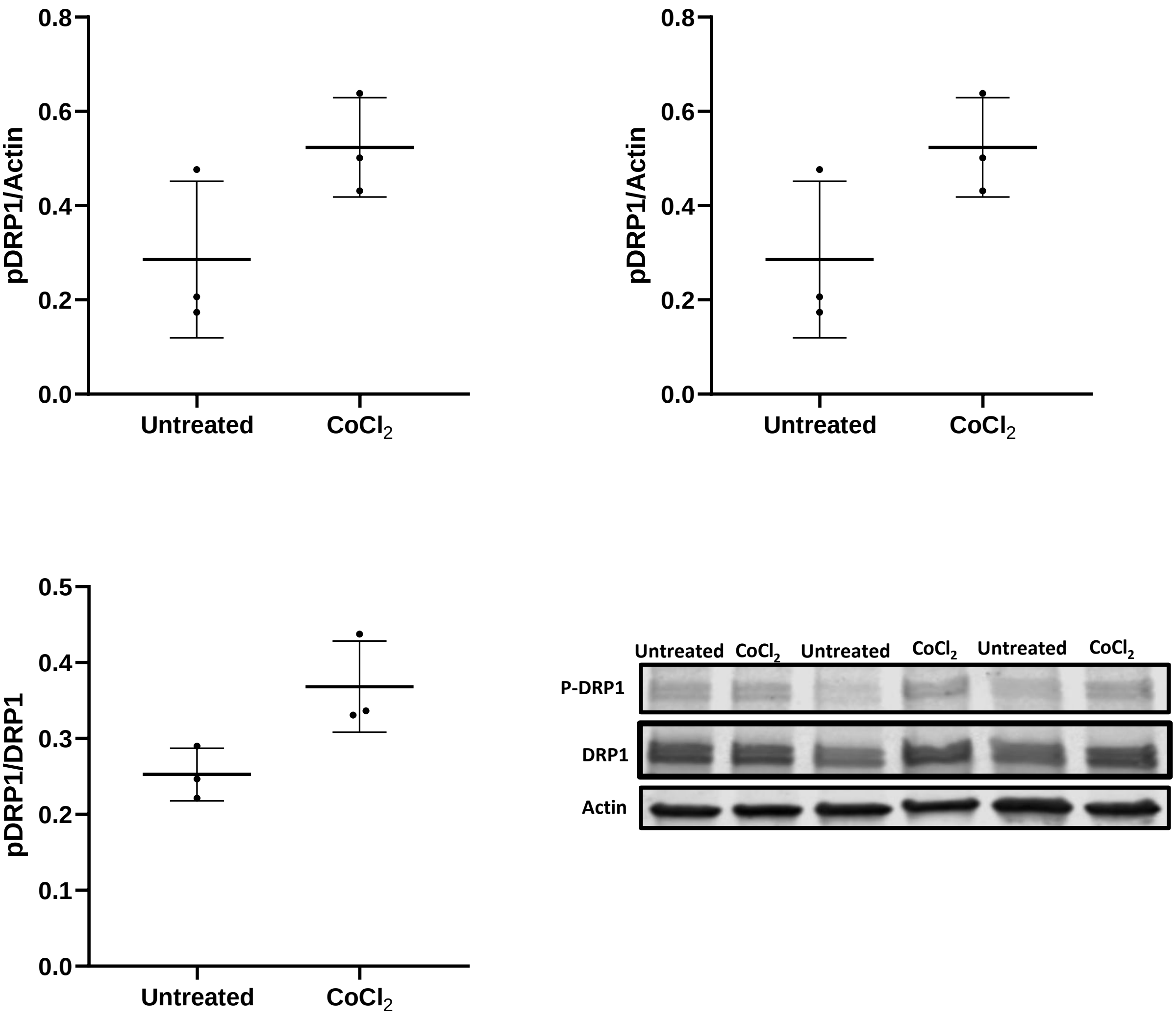

Figure S4: p-DRP1 and DRP1 expression in VCT exposed to $CoCl_2$

Immunoblot using DRP1 and p-DRP1 antibodies (S616) was performed on VCT exposed 24 h to $CoCl_2$ (200 µM). Actin was used as loading control. Graphs represent the total amount of protein relative to actin level determined by quantification of immunoblot using ImageStudiolight from LICOR. Results are represented as mean (±) SD (N = 3).
